# DDEFL1 correlated with Rho GTPases activity in breast cancer

**DOI:** 10.18632/oncotarget.22095

**Published:** 2017-10-26

**Authors:** Xiaoyun Mao, Chuifeng Fan, Xinmiao Yu, Bo Chen, Feng Jin

**Affiliations:** ^1^ Department of Breast Surgery, First Affiliated Hospital of China Medical University, Shenyang, Liaoning 110001, China; ^2^ Department of Pathology, First Affiliated Hospital and College of Basic Medical Sciences of China Medical University, Shenyang, Liaoning 110001, China

**Keywords:** DDEFL1, Rho GTPases, breast cancer

## Abstract

DDEFL1 is related to maintaining a limiting amount of ARF6 in GTP-loaded form by accelerating its GTP hydrolysis activity, which has been implicated in hepatocellular cancer pathogenesis and lung cancer development. We investigated DDEFL1 expression in breast cancer and paired normal breast tissues by immunohistochemistry and found that DDEFL1 expression was significantly associated with tumor size, lymph node metastasis, high content of elastosis and TNM stage but not with menopausal status or age. We detected the mRNA and protein expression of DDEFL1 in breast cancer cell lines by Western blotting and quantitative real-time PCR (qRT-PCR). DDEFL1 was obvious in MDA-MB-435s and MDA-MB-231 but very weak in ZR-75-1. Further experiments were conducted to evaluate the effect of DDEFL1 small interfering RNA (siRNA) transfection on the biological behavior of MDA-MB-231. After transfection, the effects of DDEFL1 inhibition on expression of mRNA and protein were also analyzed by Western blotting and qRT-PCR. Increased apoptosis and invasive ability, decreased cellular proliferation was found in MDA-MB-231 with successful DDEFL1 siRNA transient transfection (*p* < 0.05). Western blotting and qRT-PCR results showed that the DDEFL1 inhibition up-regulated Caspase-3, Apaf-1, cytochrome c, and Bax expression and down-regulated Bcl-2 expression. The DDEFL1 inhibition also down-regulated the mRNA and protein expression of Rho, CDC42 and Rac1. Our study provided a functional linkage through DDEFL1 with breast cancer biological behaviours by Rho GTPases. Possible implication of our main finding for the DDEFL1 role in breast cancer and the downstream signaling pathways for the treatment of breast cancer.

## INTRODUCTION

DDEFL1 (development and differentiation-enhancing factor-like 1), also known as UPLC1, CENTB6, ACAP4 or ASAP3(ArfGAPs with SH3 domain, ankyrin repeat, and PH domain 3), is a 903-amino acid cytoplasmic protein belonging to the subfamily of ADP-ribosylation factor (Arf) GTPase-activating proteins (GAPs) [1–3], DDEFL1 controls cell migration at least in part by destabilizing cytoskeletal protein cytoskeletal γ-actin-1 (ACTG1) [4]. A previous study indicated that it is related to maintaining a limiting amount of ARF6 in GTP-loaded form by accelerating its GTP hydrolysis activity [5, 6]. It has been implicated in hepatocellular cancer pathogenesis and lung cancer development [7, 8]. This study investigated DDEFL1 expression and its clinical relevance in breast cancer, further to analyze its role in breast cancer cells. Metastasis leads to most cancer-associated deaths, initially driven by tumor cells migration through the local environment [9]. Individual tumor cell can adopt a variety of different modes to reach their destination, all of which depend on Rho GTPases at some level. Whether DDEFL1 correlate with Rho GTPases in breast cancer cell migration?

## RESULTS

### DDEFL1 expression in invasive breast ductal cancer tissues and paired normal breast tissues

**Figure 1:**
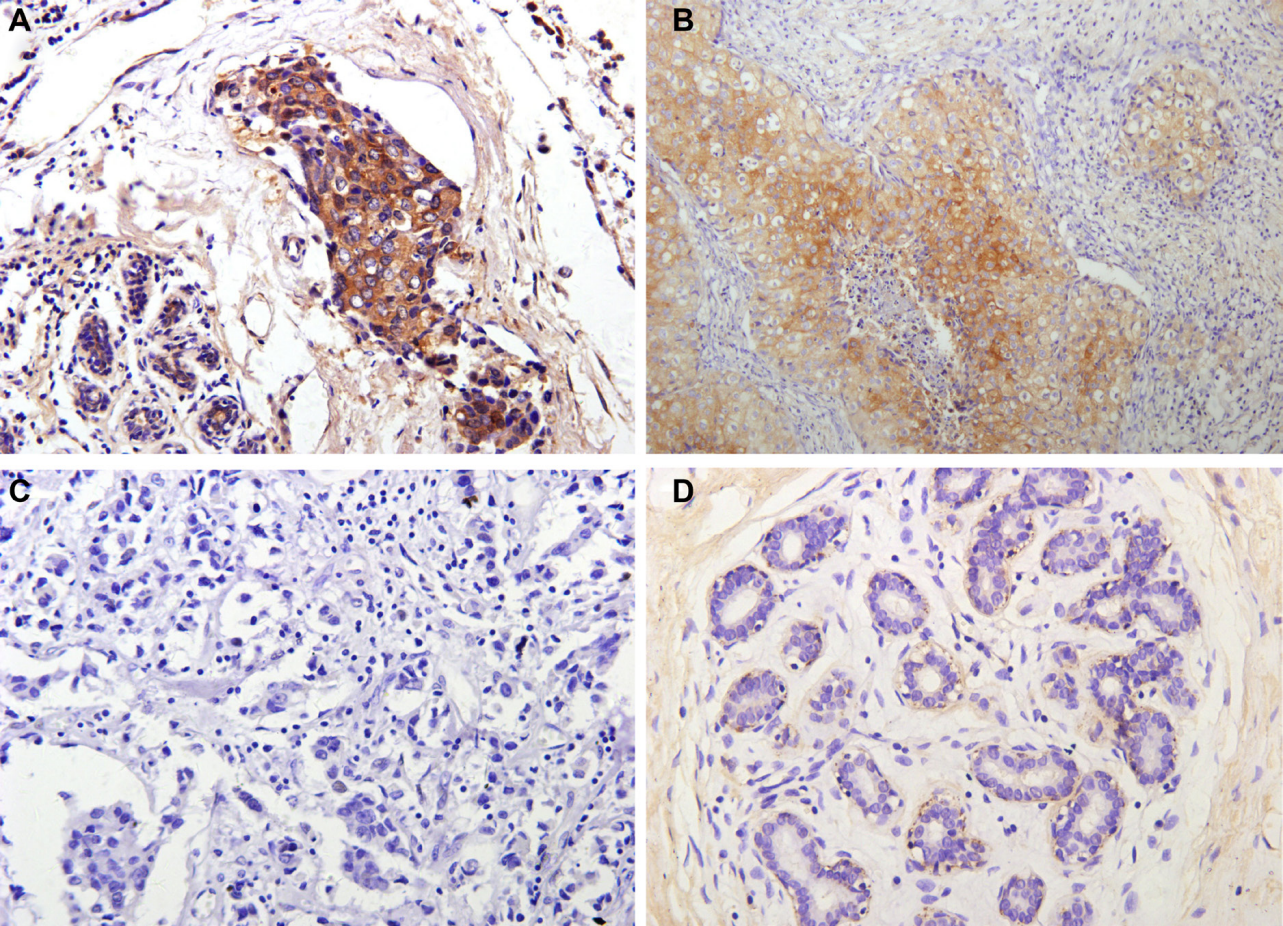
Representative immunohistochemical staining for DDEFL1 in breast cancer and paired normal breast tissue Positive DDEFL1 immunostaining was seen in invasive breast cancer (**A** and **B**), the patterns of DDEFL1 expression were cytoplasmic staining, whereas the paired normal breast tissues showed no staining of mammary epithelium (**D**). Negative DDEFL1 immunostaining was shown in cancer cells (**C**). Original magnification, all ×200.

**Figure 2:**
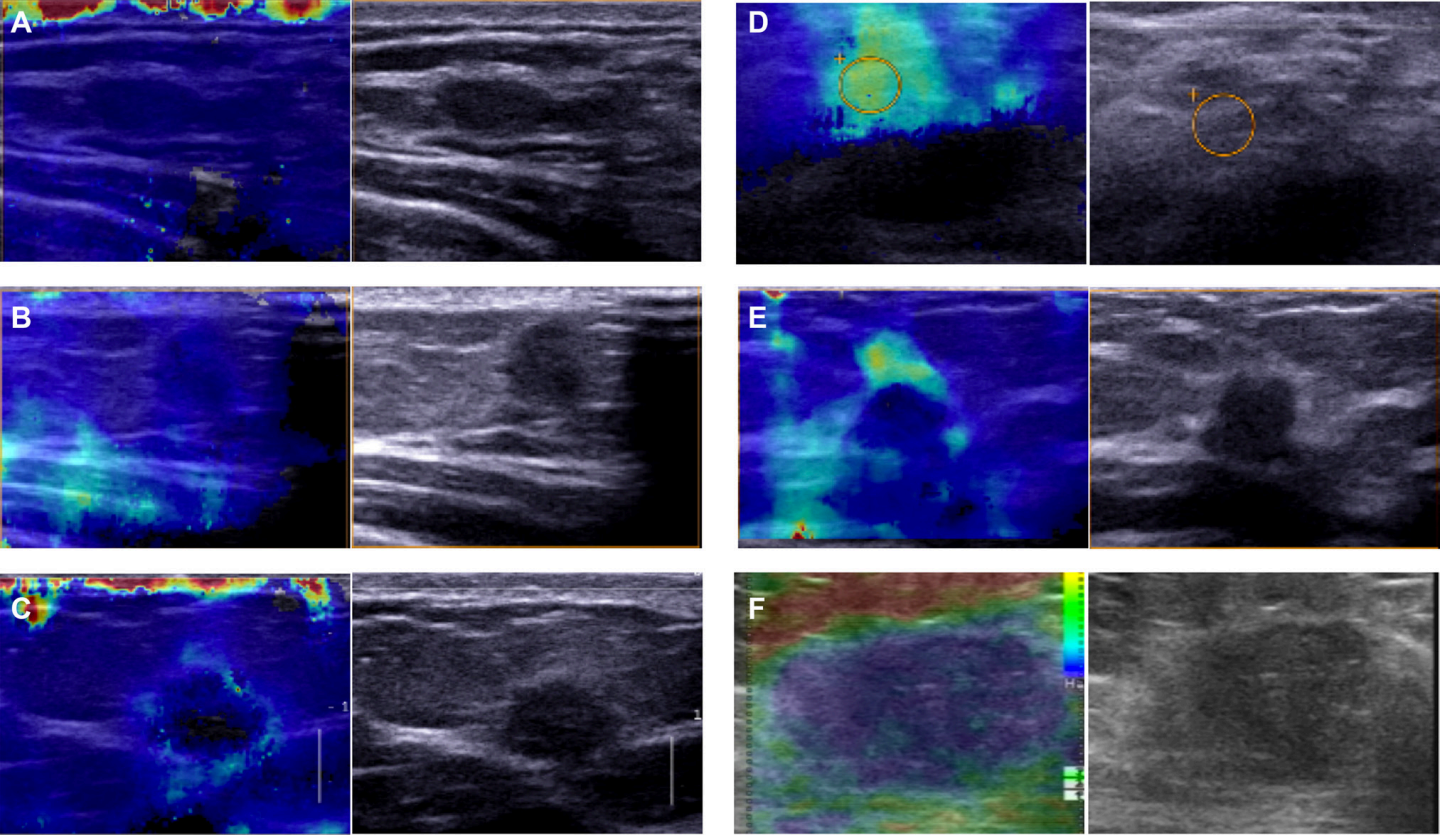
Representative pictures for the shear wave elastography score of breast cancer (**A**) The SWE image showed a case of breast cancer with SWE stiffness values of 1. On conventional B-mode US the lesion was classified BI-RADS 4B. Pathologic result: breast invasive ductal cancer. (**B**) The SWE image showed a case of breast cancer with SWE stiffness values of 2. On conventional B-mode US the lesion was classified BI-RADS 4B. Pathologic result: breast invasive ductal cancer. (**C**) The SWE image showed a case of breast cancer with SWE stiffness values of 3. On conventional B-mode US the lesion was classified BI-RADS 4B. Pathologic result: breast invasive ductal cancer. (**D**) The SWE image showed a case of breast cancer with SWE stiffness values of 4. On conventional B-mode US the lesion was classified BI-RADS 4C. Pathologic result: breast invasive ductal cancer. (**E**) The SWE image showed a case of breast cancer with SWE stiffness values of 5. On conventional B-mode US the lesion was classified BI-RADS 4C. Pathologic result: breast invasive ductal cancer. (**F**) The SWE image showed a case of breast cancer with SWE stiffness values of 5. On conventional B-mode US the lesion was classified BI-RADS 5. Pathologic result: breast invasive ductal cancer.

Immunohistochemical results revealed mainly cytoplasm staining of DDEFL1 protein in breast cancer, whereas paired normal breast tissues showed very weak or even no staining of the mammary epithelium (Figure 1). The total positive rate of DDEFL1 expression in paired normal breast tissues was 5.66% (3/53). DDEFL1 expression in breast cancer (52.33%, 101/193) was significant higher than that in adjacent normal breast tissues (*p* < 0.05). The relationship between DDEFL1 expression and different clinicopathological factors in breast cancer is shown in Table 1. DDEFL1 expression was significantly associated with tumor size, lymph node metastasis, and TNM stage but not with age or menopausal status (*p* > 0.05). The cases of DDEFL1 positive expression had mean and standard deviation values of 4.03 ± 0.93, the cases of its negative expression had mean and standard deviation values of 3.34 ± 0.99 with the Shear wave elastography score. DDEFL1 expression was significantly associated with high content of elastography (F = 4.61, *p* = 0.03). Representative pictures for the Shear wave elastography score of breast cancer were shown in Figure 2.

**Table 1:** Expression of DDEFL1 in 193 patients with breast cancer

Characteristics	*n*	Expression of DDEFL1		*p*
		Positive (*n* = 101)	Negative (*n* = 92)	
Age				χ^2^ = 2.43 (*p* > 0.05)
< 45 years	43	18	25	
≥ 45 years	150	83	67	
Menopausal status				χ^2^ = 2.83 (*p* > 0.05)
Premenopausal	60	26	34	
Postmenopausal	133	75	58	
Tumor size				χ^2^ = 7.72 (*p* < 0.01)
≤ 2 cm	91	38	53	
> 2 cm	102	63	39	
Lymph node metastasis				χ^2^ = 12.28 (*p* < 0.01)
Negative	92	36	56	
Positive	101	65	36	
Tumor stage				χ^2^ = 5.20 (*p* < 0.05)
I+II	157	76	81	
III+IV	36	25	11	

### DDEFL1 expression in ZR-75, MDA-MB-231, and MDA-MB-435s

**Figure 3:**
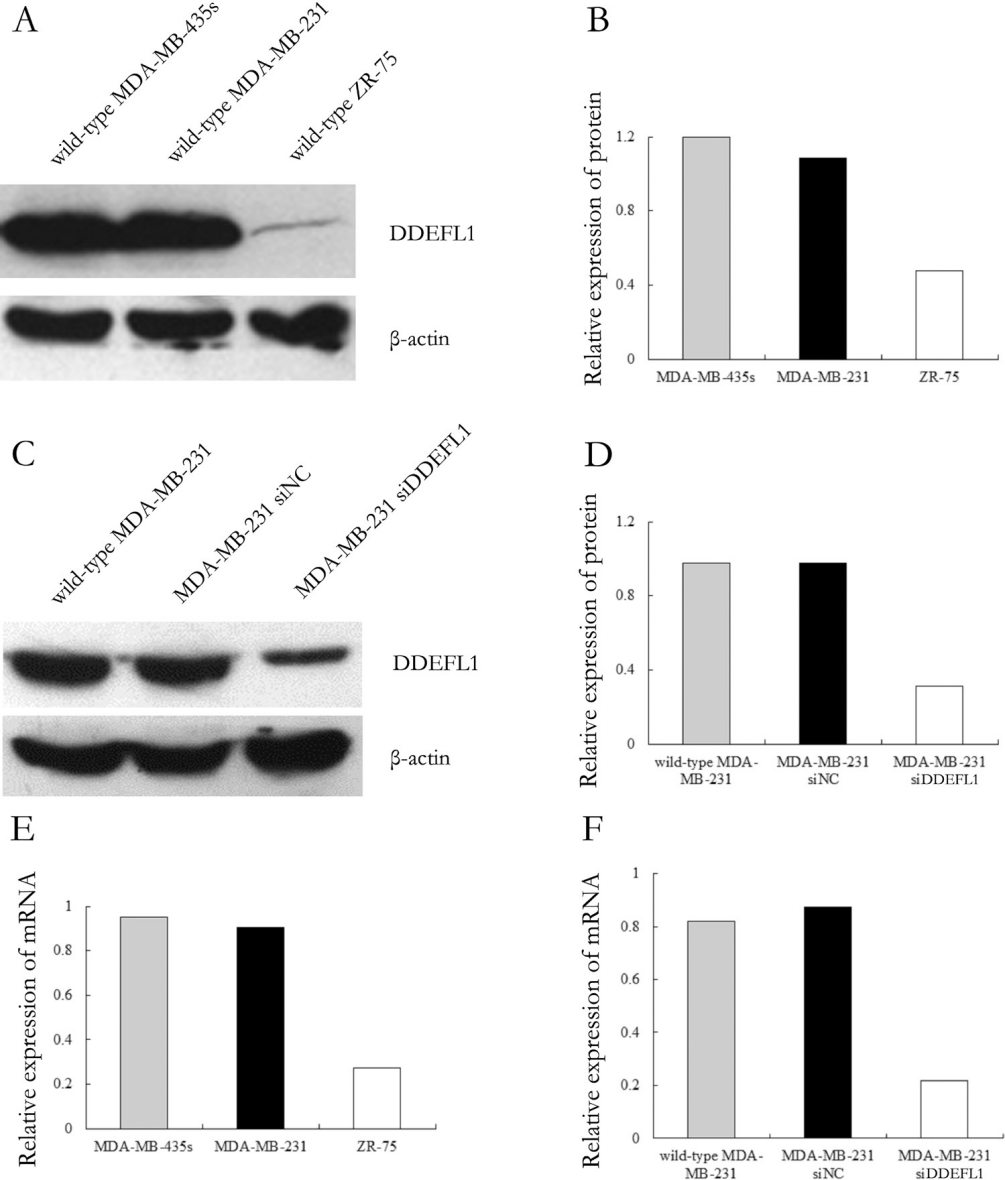
Expression patterns of DDEFL1 in breast cancer cell lines Total RNA and proteins were extracted from wild-type MDA-MB-435s, MDA-MB-231, and ZR-75 and then subjected to qRT-PCR and Western blotting for DDEFL1. Both protein (**A** and **B)** and mRNA (**E**) expression of DDEFL1 were observed consistently in MDA-MB-231 and MDA-MB-435s, but ZR-75 had very weak DDEFL1 mRNA and protein expression (A, B, and E). Therefore, we picked MDA-MB-231 for transfection with DDEFL1 siRNA. DDEFL1 protein (**C** and **D**) and mRNA (**F**) expression were obvious in the DDEFL1 siRNA-NC transfection group or wild-type MDA-MB-231 but very weak in the DDEFL1 siRNA transfection group, indicating an effective down-regulation of DDEFL1 in MDA-MB-231 by DDEFL1 siRNA.

The mRNA and protein of DDEFL1 were obvious in MDA-MB-435s and MDA-MB-231 but very weak in ZR-75 (Figure 3). Therefore, we picked MDA-MB-231 for transfection with DDEFL1 siRNA and further investigation of the role of DDEFL1 in breast cell line MDA-MB-231.

### DDEFL1 siRNA transfection in MDA-MB-231

By Western blot and qRT-PCR, DDEFL1 protein and mRNA expression was obvious in the DDEFL1 siRNA-NC transfection group or MDA-MB-231 without treatment but very weak in the DDEFL1 siRNA transfection group (Figure 3), indicating an effective down-regulation of DDEFL1 in MDA-MB-231 by DDEFL1 siRNA.

### Effect of DDEFL1 siRNA transfection on cell proliferation by MTT

**Figure 4:**
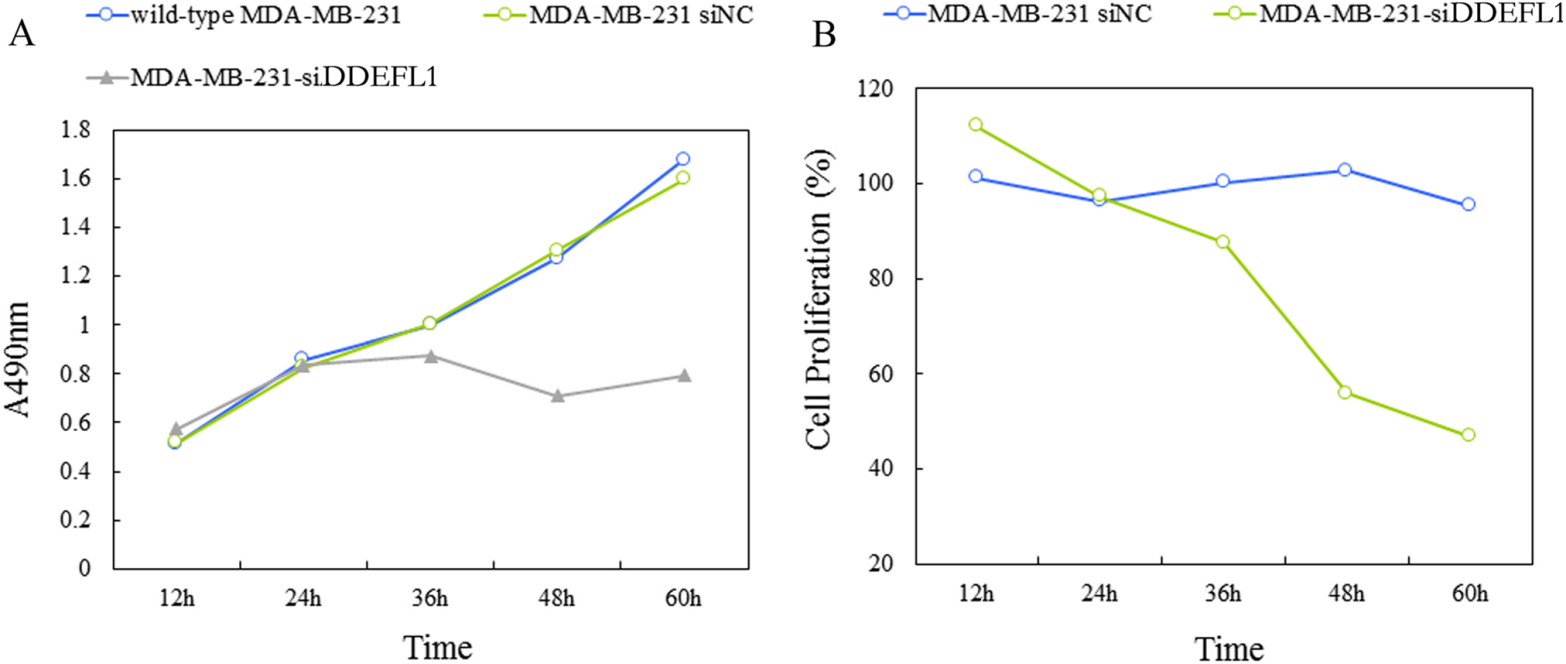
Proliferation curve of the DDEFL1 siRNA-NC transfection group and the DDEFL1 siRNA transfection group constructed by plotting absorbance against time using the MTT method The cell proliferation rate of MDA-MB-231-siDDEFL1 cells was markedly lower than that of MDA-MB-231-siNC at 48 and 60 h (*p* < 0.05). ^*^Statistically significant difference between MDA-MB-231-siDDEFL1 and MDA-MB-231-siNC (*p* < 0.05).

MTT results and the proliferation curve was shown in Figure 4, the cell proliferation rate of the DDEFL1 siRNA transfection group was markedly lower than that of the siRNA-NC transfection group or control group at 48, 60, and 72 h (*p* < 0.05), indicating that DDEFL1 siRNA induced the decreased cellular proliferation of MDA-MB-231.

### DDEFL1 siRNA transfection up-regulated the apoptosis of MDA-MB-231

**Figure 5:**
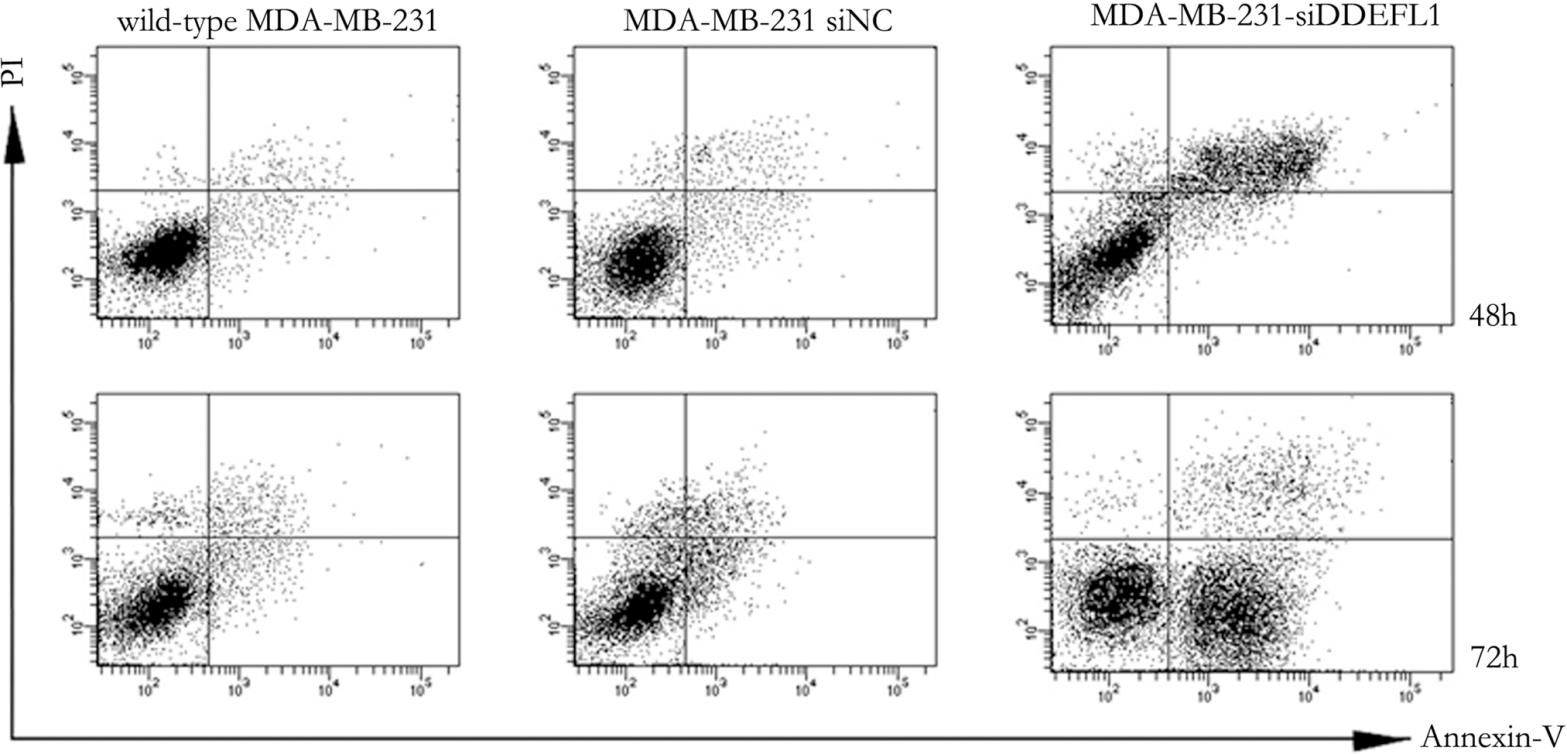
Apoptosis of MDA-MB-231-siNC, MDA-MB-231-siDDEFL1, and wild-type MDA-MB-231 as assessed by the Annexin V-FITC-labeled FACS method MDA-MB-231 transfected with siDDEFL1 showed a higher percentage of apoptosis cells than wild-type MDA-MB-231 or MDA-MB-231-siNC (*p* < 0.05).

As shown in Figure 5, MDA-MB-231 transfected with DDEFL1 siRNA showed different percentages of apoptosis with the siRNA-NC transfection group or the group of MDA-MB-231 without treatment at 48 h and 72h after transfection. The apoptotic ratio of the DDEFL1 siRNA transfection group was present at 46.8% at 48 h and 52.7% at 72 h after transfection, which was higher than the siRNA-NC transfection group (13.1% at 48 h and 10.7% at 72 h after transfection, respectively) or the group of MDA-MB-231 without treatment (15.1% at 48 h and 12.9% at 72h after transfection, respectively; *p* < 0.05).

### DDEFL1 siRNA transfection decreased the invasive ability of MDA-MB-231

**Figure 6:**
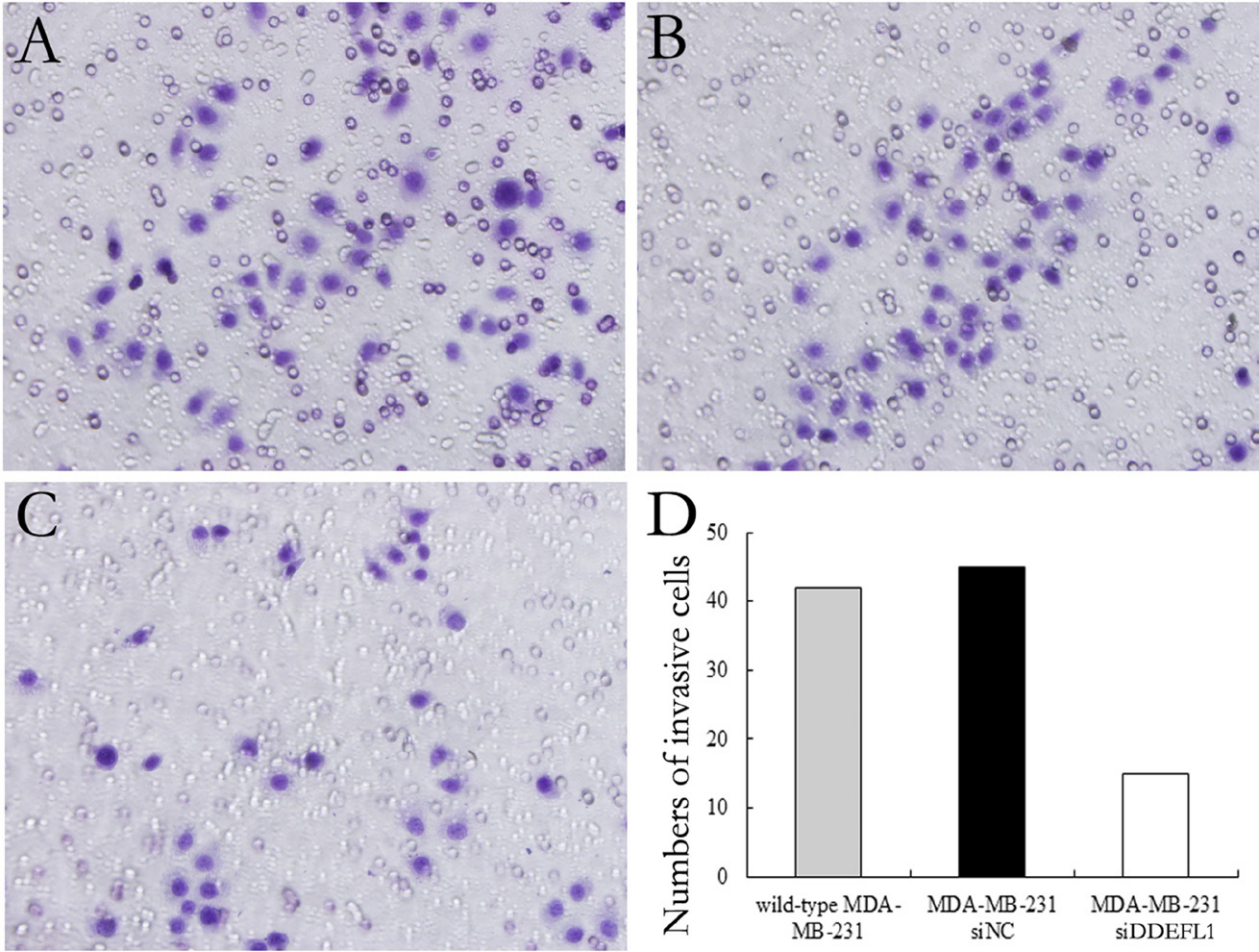
DDEFL1 siRNA transfection decreased the invasive ability of MDA-MB-231 breast cancer cells Matrigel invasion assay showed the invasive ability of wild-type MDA-MB-231 (**A**), MDA-MB-231-siNC (**B**), and MDA-MB-231-siDDEFL1 (**C**) at 48 h after transfection. The number of cells invading the lower surfaces of the filter was counted (**D**). Data represent the mean±SD of three independent experiments. ^*^p < 0.05 vs. the wild-type MDA-MB-231 group; ^#^*p* < 0.05 vs. the MDA-MB-231-siNC group.

Our results showed that the invasive ability of MDA-MB-231 was decreased with DDEFL1 siRNA transfection for 48 and 72 h compared to that in MDA-MB-231 without any additive or control cells with scrambled siRNA (*p* < 0.05; Figure 6).

### DDEFL1 siRNA transfection up-regulated the expression of apoptosis protein

**Figure 7:**
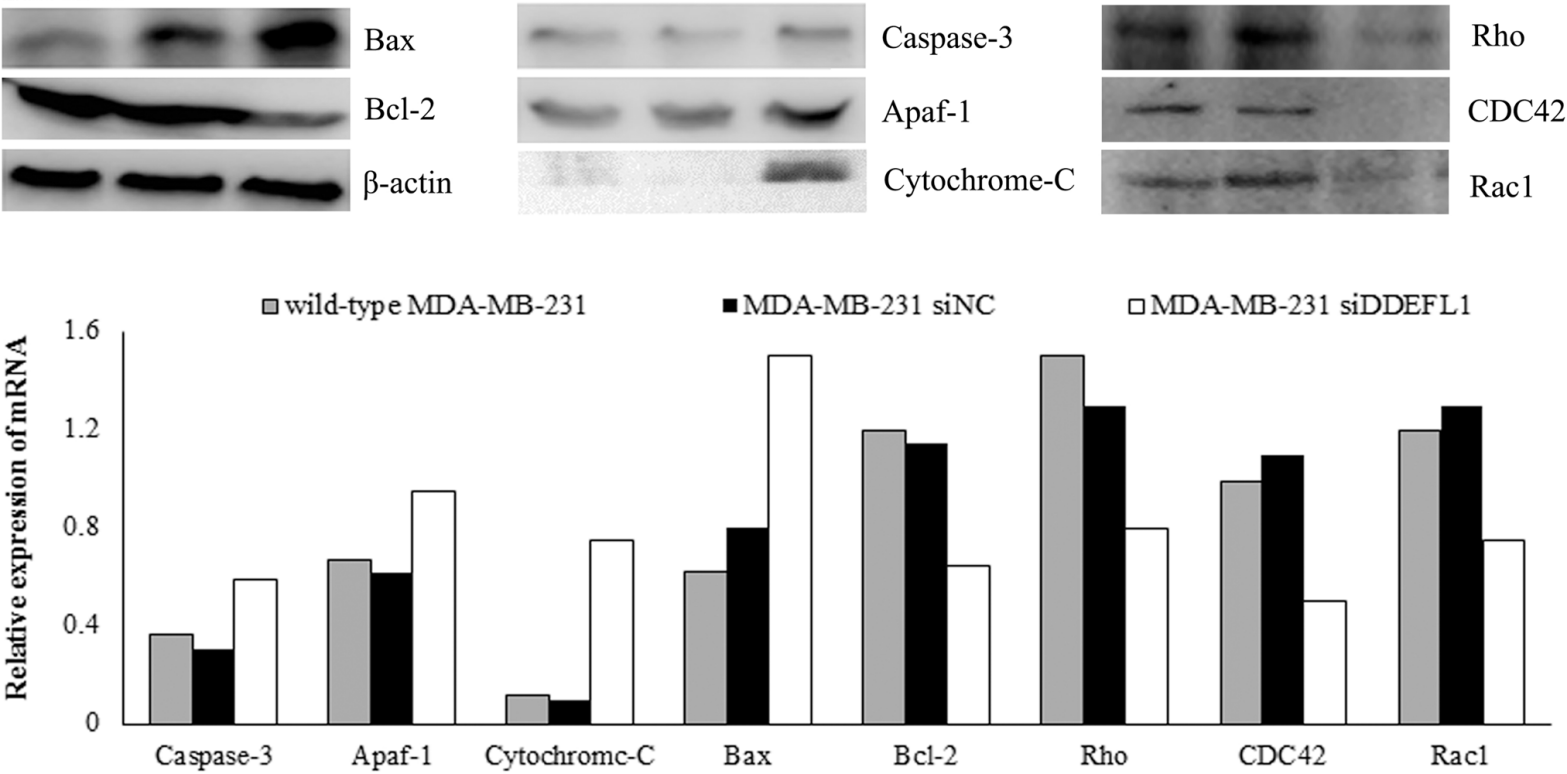
DDEFL1 siRNA transfection up-regulated the expression of apoptosis protein in MDA-MB-231 Western blotting and qRT-PCR results showed that siRNA transfection up-regulated the mRNA and protein expression of caspase-3, Apaf-1, cytochrome c, and Bax and down-regulated the mRNA and protein expression of Bcl-2. Western blotting and qRT-PCR results also showed that DDEFL1 siRNA transfection down-regulated the mRNA and protein expression of Rho, CDC42 and Rac1.

Western blotting and qRT-PCR results showed that siRNA transfection up-regulated the mRNA and protein expression of caspase-3, Apaf-1, cytochrome c, and Bax (*p* < 0.05; Figure 7) and down-regulated the mRNA and protein expression of Bcl-2 (*p* < 0.05; Figure 7). The protein levels analyzed by Western blotting also confirmed this result (Figure 7).

### DDEFL1 siRNA transfection down-regulated the expression of Rho GTPases

Western blotting and qRT-PCR results showed that siRNA transfection down-regulated the mRNA and protein expression of Rho, CDC42 and Rac1 (*p* < 0.05; Figure 7). The protein levels analyzed by Western blotting also confirmed this result (Figure 7).

## DISCUSSION

Breast cancer is the leading cause of cancer mortality among women worldwide [10, 11]. It is the most frequently diagnosed cancer and the leading cause of cancer death among females, accounting for 23% of the total cancer cases and 14% of cancer deaths in 2011 [12]. In China, breast cancer incidence increases by 3% every year, and it comprises 12.1% of the total new cancer cases and 9.6% of all deaths from breast cancer worldwide [13]. A previous research indicated that DDEFL1 is a focal adhesion-associated Arf GAP that functions in cell migration and invasion [1]. In the past decades, an insight into the DDEFL1 mechanisms of breast cancer has been developing slowly, accompanied by the development of biological technology. DDEFL1 contains an ARFGAP motif, a pleckstrin homology domain, and two ankyrin repeats [2, 14]. DDEFL1 is expressed in primary hepatocellular cancer, and DDEFL1 inhibition by siRNA transfection in hepatocellular cancer SNU475 can inhibit its proliferation [8]. Our previous study indicated that DDEFL1 expression was higher than in paired normal lung tissues and related to poor differentiation, advanced TNM stages, and positive lymph node metastasis. It contributed to the poor clinical outcome of NSCLC patients, which was possibly due to its critical roles in regulating cancer invasion [15]. How about its role in breast cancer? First, we found that DDEFL1 expression in breast cancer (63.13%, 101/160) was significantly higher than that in paired adjacent normal breast tissues (6.12%, 4/49). We found that increased DDEFL1 expression was related to lymph node metastasis and TNM stage but not with age, gender, histological subtype, tumor grade, and T stage (*p* < 0.05). We also found that DDEFL1 expression was significantly associated with high elasticity score in breast sancer. The importance of tumour stiffness in facilitating invasiveness and progression has been widely studied. A variety of molecular mechanisms related to stiffness can differentially influence breast cancer progression through complicated multiple pathways. Our result indicated that DDEFL1 may contribute to the advancement of invasive breast cancer. Therefore, we investigated DDEFL1 mRNA and protein expression in breast cancer cell lines, including MDA-MB-435s and MDA-MB-231 (high metastatic potential) and ZR-75 (low metastatic potential). RT-PCR results showed that DDEFL1 was obvious in MDA-MB-435s and MDA-MB-231 but very weak in ZR-75, which consistent with the data about DDEFL1 protein expression by Western blotting. Next, we examined DDEFL1 function in MDA-MB-435s. We found that the down-regulation of DDEFL1 had a markedly antiproliferative activity against MDA-MB-435s. To further confirm the proliferation inhibitory effects of DDEFL1 inhibition on MDA-MB-435s, FAS assay was also performed as another indicator of DDEFL1 siRNA that caused increased apoptosis to MDA-MB-435s. Moreover, compared to untreated MDA-MB-435s or MDA-MB-435s with siRNA-NC transfection, DDEFL1 inhibition up-regulated the mRNA and protein expression of caspase-3, Apaf-1, cytochrome c, and Bax. Recent studies have shown that epidermal growth factor stimulation of cells caused DDEFL1 phosphorylation at Tyr34, which promoted DDEFL1 homodimer curvature during ARF-dependent cell migration [14, 16–18]. In this study, we investigated the relationship between DDEFL1 inhibition and apoptosis. Apoptosis is the process of programmed cell death, occurs in all living organisms, and is a necessary part of the life cycle of homeostasis, organisms, and development of multiple systems, including cancer [19]. Some important molecules are involved in apoptosis, such as apoptosis-promoting molecules [cysteine containing the aspartate-specific protease (caspase) family and cytochrome c] and apoptosis-inhibitory molecules (Bcl-2 family) [20]. As revealed by Western blotting and qRT-PCR, we found that a successful DDEFL1 inhibition resulted in an increase in the mRNA or protein of apoptosis-promoting molecules such as caspase-3, Apaf-1, cytochrome c, and Bax and an increase of Bcl-2. Breast tumor progression is accompanied with the stiffness of the microenvironment [21–23]. Tumor microenvironment has also been acknowledged to contribute to tumor stiffness and breast cancer advancement. Rho family small GTPases include three well-characterized membranes, Rho, CDC42, and Rac1, all of them are important regulators of endothelial barrier properties by influencing both the endothelial actin-based cytoskeleton and the integrity of interendothelial junctions [24, 25]. Previous findings indicated Rho GTPase related with action fiber organization and have major implications in cancer stiffness [26, 27]. Our study showed that the effective DDEFL1 siRNA transfection down-regulated the mRNA and protein expression of Rho, CDC42 and Rac1. DDEFL1 is a focal adhesion-associated Arf GAP that functions in cell migration and invasion and Rho GTPases mediated the transcellular cell-ECM or cell-cell adhesions [1]. Our study provided a functional linkage through DDEFL1 with breast cancer biological behaviours by Rho GTPases. Together with the observation of the involvement of apoptosis and Rho GTPases associated factors, our results collectively unvovered DDEFL1 contribution to the tumor stiffness through Rho family small GTPases factors in breast cancer. To further clarify the role of DDEFL1 in breast carcinogenesis and advancement, the molecular mechanism of DDEFL1 should be elucidated in future studies.

### Compliance with ethical standard

All procedures performed in this study involving human participant were in accordance with the ethical standards of the institutional and approved by the Ethics Committee of China Medical University (Shenyang, China) and of the participating hospital, the First Hospital of China Medical University. The clinicopathological information of the patient was reviewed using the hospital medical records. All patients who enrolled in this study had signed informed consent form to agree to participate in this study and for the publication of the results.

## MATERIALS AND METHODS

### Patients and tissue samples

Fresh breast tissue samples, including invasive breast ductal cancer tissues and paired normal breast tissues ( > 2 cm distance from the primary cancer tissue), were collected by surgical resection at the Department of Breast Surgery, the First Affiliated Hospital of China Medical University, between January and December 2011. None of the patients underwent chemotherapy, radiotherapy, or adjuvant treatment before surgery. Patients’ ages ranged from 22 to 68 years, with an average age of 53.7 years. Each case was reviewed independently by two pathologists with a subspecialty focus in breast pathology, and only those cases wherein both pathologists finally reached a unanimous diagnosis were used. The clinicopathological information of each patient was reviewed. Shear wave elastography (SWE) is a quantitative technique that computes true tissue elasticity by measuring the velocity of shear waves as they propagate in the tissue. Shear wave propagation speed in tissue is directly determined by tissue stiffness [28–30]. The tumor elasticity was evalutated in cases with standard which had previously been reported, was graded by ultrasound from 1–5 according to SWE images [31, 32]. All patients who enrolled in this study had signed informed consent form to agree to participate in this study and for the publication of the results. And the study protocol was reviewed and approved by the Ethics Committee of China Medical University (Shenyang, China) and of the participated hospital.

### Immunohistochemical staining

Formalin-fixed, paraffin-embedded specimens were cut into 4-μm-thick sections, which were subsequently de-waxed and hydrated. The immunohistochemical staining for DDEFL1 monoclonal antibody (sc-135740; 1:50; Santa Cruz) was performed using UltraSensitive™ S-P kits (Maixin-Bio, China) according to the manufacturer's protocols. For the negative control, phosphate-buffered saline (PBS) was used in place of the primary antibodies. We adopted the German semi-quantitative score system in considering the staining intensity and area extent, which has been widely accepted and published in previous studies [33–35]. Every lesion was given a score according to the intensity of the staining (0 = no staining, 1 = weak staining, 2 = moderate staining, 3 = strong staining) and the extent of stained cells (0 = 0%, 1 = 1–10%, 2 = 11–50%, 3 = 51–80%, 4 = 81–100%, negative = 0% area staining). The final immunoreactive score was determined by multiplying the intensity scores with the extent of positivity scores of stained cells, with the minimum score of 0 and a maximum score of 12 [33–35]. Slides were independently examined by two pathologists (C.F. Fan and J.H. Yu). If there was a discrepancy in individual scores, both pathologists reevaluated the scores by reaching a consensus before combining the individual scores. To obtained statistical results, a final score of ≤ 1 was considered as negative, whereas scores of ≥ 2 were considered as positive.

### Breast cancer cell lines and cell culture conditions

Breast cancer cell lines MDA-MB-435s and MDA-MB-231 (high metastatic) and ZR-75 (low metastatic) were chosen for our study and maintained under recommended culture conditions. These cell lines were obtained from the American Tissue Culture Collection (Manassas, VA, USA) and was stored in the laboratory of Pathology, First Affiliated Hospital and College of Basic Medical Sciences of China Medical University (Shenyang, China). All cells were cultured in RPMI 1640 medium (Gibco, USA) or DMEM (Gibco, USA) supplemented with 10% fetal bovine serum (FBS; Hyclone, USA) in a 5% CO_2_ humidified atmosphere at 37°C.

### Western blotting of DDEFL1

ZR-75, MDA-MB-231, and MDA-MB-435s breast cells were washed with ice-cold PBS and then lysed in lysis buffer containing 10 mM Tris (pH 7.5), 150 mM NaCl, 10 mM ethylenediaminetetraacetic acid (EDTA), 1% sodium dodecyl sulfate (SDS), 1 mM sodium orthovanadate, and a mixture of protease inhibitors (1 mM phenylmethylsulfonyl fluoride, 1 μg/mL pepstatin A, and 2 μg/mL aprotinin). The lysates were sonicated for 10 s, centrifuged for 20 min at 20,000 × g, and then stored at −70°C. Equal amounts (25 μg) of the cell lysates were resolved by 12% SDS-PAGE and transferred to polyvinylidene fluoride membranes. After blocking, blots were incubated mouse anti-DDEFL1 monoclonal antibody (sc-135740; 1:500; Santa Cruz Biotechnology) or b-actin (1:1000; Zhongshan Golden Bridge Biotechnology) overnight at 4°C followed by each corresponding second antibody at room temperature for 1 h at 37°C. The results developed by ECL (Pierce Biotechnology, USA). The protein bands were then analyzed using the BioImaging System (UVP, USA). The grayscale values of DDEFL1 were normalized to the values of the corresponding b-actin band to determine the expression level of the protein. The experiments were repeated at least three times independently.

### Quantitative real-time PCR (qRT-PCR) of DDEFL1

Total RNA from ZR-75-1, MDA-MB-231 and MDA-MB-435s was extracted with Trizol reagent (Invitrogen, Carlsbad, CA, USA). Reverse transcription was performed using the RNA PCR Kit (AMV ver. 3.0, Takara, Japan) according to the manufacturer's protocols. qRT-PCR was performed using the SYBRÒ Premix Ex Taqä II Kit (Takara) using the 7500 Real-time PCR System (Applied Biosystems, USA). Template cDNA (2 μl) was added to the final volume of 20 μl of reaction mixture. The sequence of primers used for RT-PCR analysis is provided in Table 2. The expression of the selected genes was normalized to GADPH, which was used as an internal housekeeping control. Our results showed that the mRNA and protein of DDEFL1 was obvious in MDA-MB-231 and MDA-MB-435s, but very weak in ZR-75-1. Therefore, we picked MDA-MB-231 for transfection with DDEFL1 small interfering RNA (siRNA).

**Table 2:** Sequence of primers used for RT-PCR analysis used in the study

	Primer (forward)	Primer (reverse)	Product size (bp)
GADPH	CCACCCATGGCAAATTCCCATGGCA	TCTAGACGGCAGGTCAGGTCCACC	597
DDEFL1	ACCTCAGCTAGTGACGTATGG	CGGAGTCCCAGGACACTGTG	492
Caspase-3	TGTTTGTGTGCTTTGAGCC	CACGCCATGTCATCATCAAC	210
Bcl-2	GGATTGTGGCCTTCTTTGAG	CCAAACTGAGCAGAGTCTTC	230
Apaf-1	TTAGGAGCCAGGTGCGGT	GCTTGTCTTTCTTCCCATTTTTC	148
Cytochrome c	GAGCGGGAGTGTTCGTTGT	GTCTGCCCTTTCTTCCTTCT	327
Bax	CCCGAGAGGTCTTTTTCC	GCCTTGAGCACCAGTTTG	108

### DDEFL1 siRNA transfection

The human DDEFL1 gene sequence was obtained from GenBank. According to the design principle of siRNA, siRNA Target Designers (Ambion) was used to design siRNAs targeting the specific DDEFL1. The sequence was synthesized by Shanghai Biochemical Engineering Co., Ltd. MDA-MB-231 was cultured in a 24-well plate for 24 h before the experiment of transient transfection. Cells were transfected with Lipofectamine 2000 (Invitrogen) according to the manufacturer's instruction. After transfection, cells were harvested at 48 h to measure the protein levels. Control DDEFL1 siRNA (sc-88264; 1:500; Santa Cruz Biotechnology) and negative control siRNA (sc-36869; 1:500; Santa Cruz Biotechnology) were used in Western blotting to verify the down-regulation of DDEFL1. The control study using nonspecific siRNA was carried out under the same condition.

### 3-(4,5-Dimethylthiazol-2-yl)-2,5-diphenyltetrazoliumbromide (MTT) assay

Cells were divided into groups as follows: DDEFL1 siRNA transfection group and siRNA-NC (negative control) transfection group. MDA-MB-231 without treatment served as control. The cell proliferation of each group was assessed at various time points using the MTT assay. Briefly, 2000 cells were seeded in each well of a 96-well plate and were allowed to adhere for 8 h. Then, 5 mg/ml MTT (Sigma, Germany) was added to each well and was incubated for 4 h. Cells were lysed by adding 150 μl/well dimethylsulfoxide and read at 490 nm absorbance wavelength in a microplate reader.

### Flow cytometry apoptosis assay

The flow cytometry apoptosis analysis was assessed by an Annexin V-FITC/propidium iodide (PI) double staining kit (Genmed Bioscience, China) following the manufacturer's instructions. Each group was plated in six wells. Cells were continuously cultured for 48h and 72 h and then harvested. Before reading on the flow cytometer, cell suspensions were resuspended with a 1 × binding buffer, and exposed to 5 mL Annexin V-FITC (20 μg/mL) and 10 mL PI (50 μg/mL). After incubation for 20 min in the dark, the samples were subjected to FACSCan flow cytometry (equipped with CellQuest and ModFITLT for Mac V1.01 software; Becton Dickinson, San Jose, CA, USA).

### Matrigel invasion assay

Cell invasive ability was analyzed with a 24-well Transwell with 8.0-μm pore polycarbonate membrane inserts (Corning, Inc., NY, USA) according to the manufacturer's instructions. Matrigel was applied to the upper surface of the membranes. After transfection for 48h and 72 h, cells of each group were seeded on the upper chamber (5 × 10^4^ cells/well) and incubated for 18 h. Cells which invaded the surface of the membrane were fixed with methanol and stained with hematoxylin. Cells that invaded and moved onto the lower surface of the filter membrane were counted in 10 random high-power fields (400 × ) by microscope. The experiment was repeated five times. Data was shown in mean ± standard deviation (SD).

### Western blotting and qRT-PCR analyses of apoptosis

The expression of apoptosis protein of each group was analyzed by Western blot as previous described using specific antibody against Caspase-3 (sc-271759, Santa Cruz, 1:100), Bcl-2 (sc-7382, Santa Cruz, 1:250), Apaf-1 (sc-135625, Santa Cruz, 1:500), Cytochrome C (sc-13156 Santa Cruz, 1:200), Bax (sc-70406, Santa Cruz, 1:200) or β-actin (Zhongshan Golden Bridge Biotechnology, 1:1000). The apoptosis protein mRNA was detected by qRT-PCR as previous described. Primers used for qRT-PCR analysis were provided in Table 1. The experiments were repeated at least three times independently.

### Western blotting and qRT-PCR analyses of Rho, CDC42, and Rac1

Rho family small GTPases include three well-characterized membranes, Rho, CDC42, and Rac1, have emerged as key regulators acting antagonistically to regulate endothelial barrier function by influencing both the endothelial actin-based cytoskeleton and the integrity of interendothelial junctions. The protein Rho, CDC42, and Rac was detected by Western blotting in the DDEFL1 siRNA transfection group and siRNA-NC (negative control) transfection group. Wild-type MDA-MB-231 without treatment served as the control group. Western blot was performed as previously mentioned using primary antibodies against Rho (sc-4418, Santa Cruz, 1:500), CDC42 (sc-8401, Santa Cruz, 1:500), and Rac1 (sc-514583, Santa Cruz, 1:100). The mRNA of Rho, CDC42, and Rac1 was detected by qRT-PCR as previously mentioned procedure in the DDEFL1 siRNA transfection group and siRNA-NC (negative control) transfection group. Wild-type MDA-MB-231 without treatment served as the control group.

### Statistical analysis

SPSS version 13.0 for Windows was used for all analyses. Pearson's χ^2^ test was used to analyze the relationship between DDEFL1 expression and the clinicopathological factors in breast cancer. One-way analysis of variance was performed to compare data from the densitometry analysis of FAS, MTT, Western blotting and RT-PCR, and Matrigel invasion assay. The statistical significance in this study was set at *p* < 0.05. All reported *p*-values are two-sided.
